# Long-term memory T cells as preventive anticancer immunity elicited by TuA-derived heteroclitic peptides

**DOI:** 10.1186/s12967-021-03194-6

**Published:** 2021-12-24

**Authors:** Angela Mauriello, Beatrice Cavalluzzo, Carmen Manolio, Concetta Ragone, Antonio Luciano, Antonio Barbieri, Maria Lina Tornesello, Franco M. Buonaguro, Maria Tagliamonte, Luigi Buonaguro

**Affiliations:** 1 Lab of Innovative Immunological Models, Istituto Nazionale Tumori - IRCCS “Fondazione Pascale”, Via Mariano Semmola, 52, 80131 Naples, Italy; 2 Animal Facility, Istituto Nazionale Tumori - IRCCS “Fondazione Pascale”, Naples, Italy; 3 Mol Biol and Viral Oncogenesis, Istituto Nazionale Tumori - IRCCS “Fondazione Pascale”, Naples, Italy

**Keywords:** Tumor antigens, Heteroclitic peptides, Viral antigens, Cross-reacting TCRs, Cancer vaccines

## Abstract

The host’s immune system may be primed against antigens during the lifetime (e.g. microorganisms antigens—MoAs), and swiftly recalled upon growth of a tumor expressing antigens similar in sequence and structure. C57BL/6 mice were immunized in a preventive setting with tumor antigens (TuAs) or corresponding heteroclitic peptides specific for TC-1 and B16 cell lines. Immediately or 2-months after the end of the vaccination protocol, animals were implanted with cell lines. The specific anti-vaccine immune response as well as tumor growth were regularly evaluated for 2 months post-implantation. The preventive vaccination with TuA or their heteroclitic peptides (hPep) was able to delay (B16) or completely suppress (TC-1) tumor growth when cancer cells were implanted immediately after the end of the vaccination. More importantly, TC-1 tumor growth was significantly delayed, and suppressed in 6/8 animals, also when cells were implanted 2-months after the end of the vaccination. The vaccine-specific T cell response provided a strong immune correlate to the pattern of tumor growth. A preventive immunization with heteroclitic peptides resembling a TuA is able to strongly delay or even suppress tumor growth in a mouse model. More importantly, the same effect is observed also when tumor cells are implanted 2 months after the end of vaccination, which corresponds to 8 – 10 years in human life. The observed potent tumor control indicates that a memory T cell immunity elicited during the lifetime by a antigens similar to a TuA, i.e. viral antigens, may ultimately represent a great advantage for cancer patients and may lead to a novel preventive anti-cancer vaccine strategy.

**Supplementary Information:**

The online version contains supplementary material available at 10.1186/s12967-021-03194-6.

## Introduction

Tumor antigens (TuAs) expressed by cancer cells can be classified in tumor-associated antigens (TAAs), over-expressed self-antigens shared among patients with the same malignancy [1], and tumor-specific antigens (TSAs), mutated neoantigens specific for each patient [2]. However, most of the numerous clinical trials evaluating therapeutic cancer vaccines based on TAAs or TSAs have failed to successfully contain either tumor growth or disease progression [3]. Indeed, a prolonged tumor growth at primary site represents a driving factor for the formation of the TME by release of soluble factors (e.g. chemokines and cytokines) recruiting immune suppressive cell populations [4]. Therefore, targeting and eliminating cancer cells in the very early stages (Tis or T0), when the TME is not yet or about to be formed, would be the only possibility for the immune system to be effective against the tumor. However, to achieve such a potent anti-tumor effect, a memory CD8^+^ T cell immunity specific for TuAs should be already established and prompt to expand upon development of cancer cells.

Such an anti-TuA memory T cell immunity can only be elicited by a preventative vaccine which is not available and will likely never be. Nevertheless, every individual is exposed to intracellular pathogens (i.e. viruses and intracellular bacteria) and intestinal microbiota, collectively microorganisms (MOs), which enter the body during the host’s lifetime. Altogether, MOs are a natural source of non-self antigens expressed by host’s cells in the context of the HLA class I molecules, inducing a wide pool of specific memory CD8^+^ T cell clones specific for the MOs’ antigens (MoAs). The latter memory CD8^+^ T cell clones may turn out to be a natural “anti-cancer vaccination” if a nascent tumor lesion should contain cancer cells accidentally expressing TuAs similar or identical to MoAs [5]. Indeed, the host’s immune system would promptly expand to target and clear cancer cells before a larger tumor and an immunosuppressive TME is formed. In other words, this condition would stop the cancer immunoediting process at the Elimination stage, preventing the tumor from entering the Equilibrium and the Escape stages (the three Es of the cancer immunoediting) [6].

The improved prognosis in cancer patients bearing tumors expressing TuAs sharing sequence homology with viral antigens has been anecdotally reported in melanoma and pancreatic cancer [7, 8]. Moreover, our group has shown very recently that several TAAs share sequence and structural homology with viral antigens [9] and that liver cancer cells presenting a mutated TSA mimicking the Vaccinia Virus (VV) antigen can be identified in a long-term survival patient with hepatocellular carcinoma (HCC) [10]. Finally, a decreased tumor growth and extended survival has been shown in a melanoma animal model presenting a TuA sharing sequence homology to a commensal’s antigen [11].

The efficacy of memory CD8^+^ T cell clones specific for MoAs as a natural “anti-cancer vaccination” can be predicted even if the TuA shows mismatches with the MoA. This is possible because a single T cell receptor (TCR) shows a cross-reactivity against similar antigens and may recognize at least 10^6^ different MHC-bound peptides[12]. Such TCR degeneracy gives rise to a repertoire of highly cross-reactive clonal T cells responding to an array of epitopes similar to a given non-self pMHC complex [13, 14]. This reduces the possibility of expansion for mutant escape variants recognized by different TCRs specific for the original pMHC [15, 16]. Indeed, the epitope binds to the HLA molecule with specific residues in fixed positions along the sequence (anchor residues) and only the central residues are exposed to bind the TCR (http://www.cbs.dtu.dk/services/NetMHC/logos.php) [17, 18]. Therefore, two unrelated antigens sharing the same TCR-facing central residues, or showing conservative variations at those positions, are very likely recognized by the same TCRs even if the peripheral residues of the epitope are different, without affecting the structural conformation of the entire epitope.

In the present study we aimed at verifying whether an established T cell memory specific for antigens sharing sequence and structural similarity with a TuA may control tumor growth in an animal model. Given that a MoA with such characteristics of similarity to the TuAs HPV E7 and Trp2 antigens is not available, heteroclitic peptides (hPep) were designed from the TuA sequences in order to simulate a MoA. Heteroclitic peptides are sequence variants of a wild type peptide that should presumably stimulate stronger T cell responses [19]. Indeed, the amino acid (aa) substitutions should increase peptide antigenicity and immunogenicity by enhancing peptide-binding affinity for human histocompatibility-linked leukocyte antigens (HLA) and/or improving T cell receptor (TCR) recognition of the bound peptide [20]. In particular, hPep have been previously reported to elicit T cells cross-reacting with wt epitopes, able to control tumor growth [21]. On the contrary, the ability of hPep to elicit a long-term memory T cell response as preventive anti-cancer immunity has never been shown before.

The results showed that the preventive vaccination with TuA or hPep was able to delay (B16) or completely suppress (TC-1) tumor growth when cancer cells were implanted immediately after the end of the vaccination. More importantly, TC-1 tumor growth was significantly delayed, and suppressed in 6/8 animals, also when cells were implanted 2-months after the end of the vaccination. The observed potent tumor control indicates that a memory T cell immunity elicited during the lifetime by an antigen similar to a TuA is cross-reactive with the wt TuA. This may ultimately represent a great advantage for cancer patients and may lead to a novel preventive anti-cancer vaccine strategy.

## Materials and methods

### BLAST homology search

The HPV-E7 and Trp2 TAAs were submitted to BLAST for a protein homology search against viral (Viruses—taxid:10,239) and microbiota (Bacteroidetes—taxid:976; Firmicutes—taxid:1239) sequences within the non-redundant protein sequences database (https://blast.ncbi.nlm.nih.gov/Blast.cgi). Homologous microorganisms’ protein sequences have been extracted from the protein database of the National Center for Biotechnology Information (NCBI) (https://www.ncbi.nlm.nih.gov/) and epitope prediction has been performed with the NetMHCstabpan 1.0 tool.

### Prediction and design of heteroclitic peptides

Heteroclitic peptides (hPep) for Trp2 and HPV E7 antigens were designed introducing each of the 20 aminoacids at position 3, 4 or 5 of the peptides. The predicted binding affinity of each heteroclitic peptide to the H2-Db was assessed by the NetMHCpan version 4.1 algorithm.

### Peptide synthesis

Individual peptides were synthesized at a purity > 95%. Lyophilized powder was dissolved in dimethylsulfoxide (DMSO; Sigma-Aldrich), diluted in phosphate-buffered saline (1 × PBS; Gibco Life Technologies) and stored at − 80 °C until use.

### Molecular docking of the E7 wt and Trp2 peptides and their heteroclitc variants with the H-2 class I histocompatibility antigen H-2Db

To perform molecular docking analysis of the complex between the major histocompatibility complex class I (MHC-I) H-2Db and the HPV-16 E7, Trp2 peptides and their heteroclitic variants, the structural data available for H-2Db was searched in the Protein Data Bank (PDB) (https://www.rcsb.org/). Although several structure of this murine MHC-I complexed with variety of peptides have been reported, no structural data is available for the specific H-2Db/HPV-16-E7 as well as for H-2Db/Trp2 complexes. The PDB entry 1FG2 was selected for the analysis, corresponding to a complex of H-2D^b^ with a peptide with sequence KAVYNFATC [22]. The molecular modelling and docking analysis was performed by PyMOL and MolSoft molecular graphics systems.

### Cell line and mice

C57BL/6 (H-2-Db) female mice, 8 weeks old, were purchased from Harlan (Udine, Italy). All animals were housed at the Animal Facility of the Istituto Nazionale Tumori “Pascale” (Naples, Italy). Mice were housed in number of 2–3 per cage and maintained in a conventional facility on a 12 h light:12 h dark cycle (lights on at 7:00 a.m.) in a temperature-controlled room (22 ± 2 °C) and with food and water ad libitum at all times. The experimental protocols were in compliance with the European Communities Council directive (86/609/EEC).

Mouse melanoma B16F10 (ATCC, CRL-6323) cells were cultured in DMEM medium supplemented with 10% heat inactivated FBS, 100 U/ml penicillin and 100 mg/ml streptomycin (Invitrogen, Carlsbad, CA) at 37 °C with 5% CO2. Cells were tested for mycoplasma before inoculation in mice (ATCC®, 30-1012 K™). Mouse lung TC-1 tumor cells expressing HPV16 E7 protein (ATCC, CRL-2493), were cultured in RPMI medium supplemented with 10% heat inactivated FBS, 100 U/ml penicillin and 100 mg/ml streptomycin (Invitrogen, Carlsbad, CA), HEPES 10 mM, MEM 0.1 mM and Na Piruvate 1 mM at 37 °C with 5% CO2.

### Animal experiments and immunizations

B16F10 and TC1 cells were harvested in exponential growth phase by trypsinization and washed twice with ice-cold PBS. C57BL/6 mice were subcutaneously injected with 5 × 10^4^ and 1 × 10^5^ cells/mouse of B16F10 or TC1 respectively on the right back flank. A cell aliquot (200μL) containing 1 × 10^6^ cells was loaded in 21G syringes for intra-splenic injection. The tumor size was measured and documented every two days with a caliper, starting on day 7, and calculated using the formula (AxB^2^)/2 (A as the largest and B is the smallest diameter of tumor). Tumor growth was documented as mean tumor size with standard error. To record the survival of the tumor-bearing mice, either natural death or a tumor diameter greater than 1500 mm^3^ leading to death was counted as death.

### Drugs administration

Cyclophosphamide (CTX) (Endoxan®, Baxter) (10 mg/Kg) and Paclitaxel (PTX) (Taxol®, BMS) (5 mg/Kg) diluted with phosphate-buffered saline (PBS) were administered via intraperitoneal injection (i.p.). The dose was extrapolated to human equivalent dose (HED) [23]. Metronomic Chemotherapy was weekly administered one day before the vaccine administration until the end of the experiment. An anti-mouse PD-1 MAb (BioXCell, West Lebanon, NH USA) was used as checkpoint inhibitor (CI) and administered via intraperitoneal injection (i.p.) at a dose of 100 μg.

### Preventive immunization experiment

**Fig. 1:**
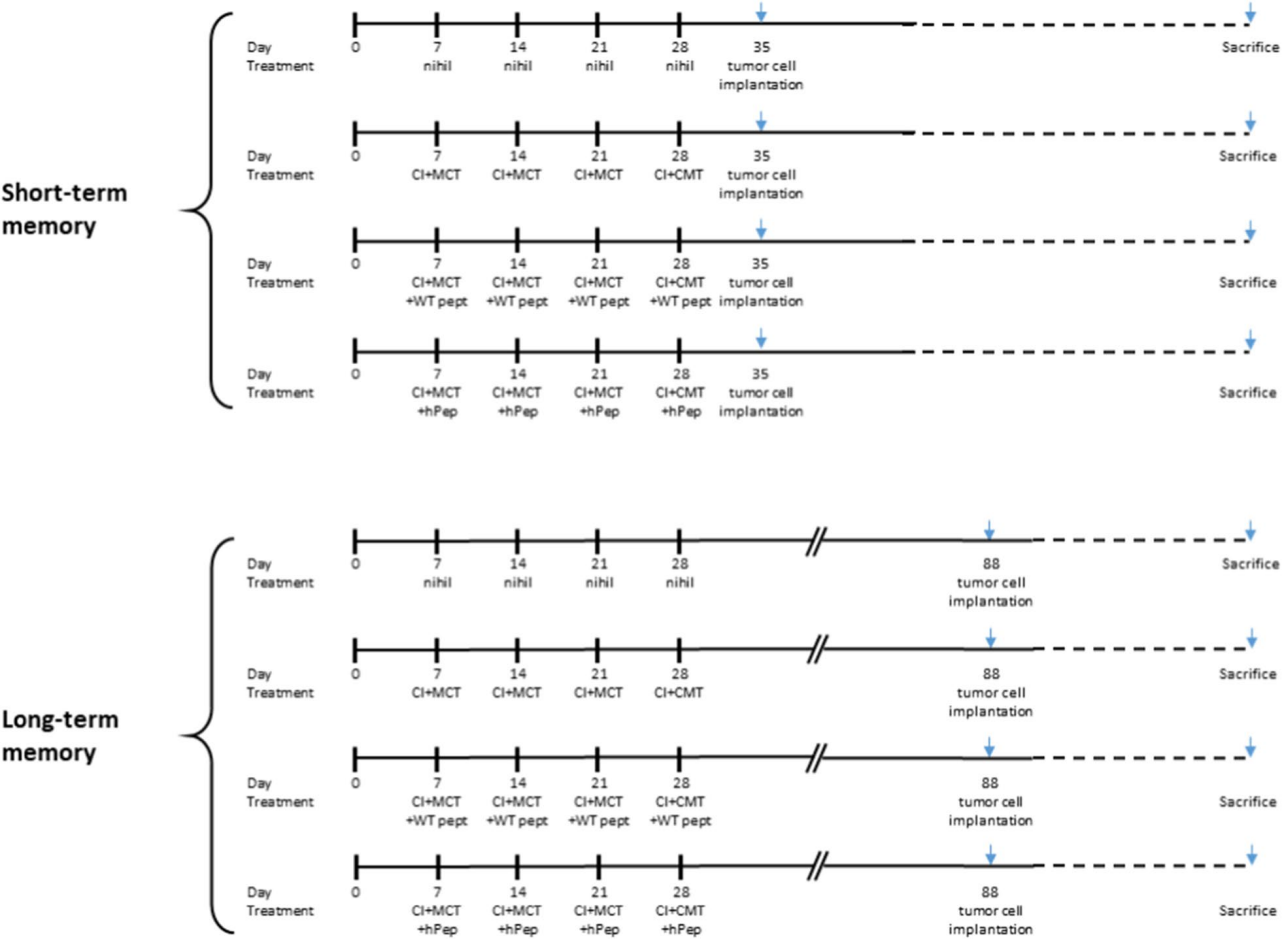
Experimental animal protocol. The short-term and long-term immunization protocols included 4 experimental groups (C57BL/6 mice). The first one is the PBS control (nihil); the second one is treated only with checkpoint inhibitor and metronomic chemotherapy (CI + MCT); the third is the combination with the wt peptide (E7 or Trp2); the fourth is the combination with the mix of heteroclitic peptides (hPep). Tumor cell implantation is either the B16 or the TC1 cell lines

Preventive experimental setting was performed as follow: C57BL/6 mice were immunized once a week for 4 weeks by subcutaneous administration (s.c.) with WT 100ug or a mix of equimolar ratio of heteroclitic peptides (100 ug tot) formulated in 50ug PolyI:C. At the end of the immunization protocol, a group of animals were implanted with of B16F10 or TC1 cell lines (short-term memory group). In parallel, a second group was left for 2 months without any further treatment before implantation of the same number of cells (long-term memory group). Immunizations were performed in combination with metronomic chemotherapy (MCT) and CI [24–26]. One group was treated only with MCT and CI (MCT + CI group). Control mice were treated with endotoxin-free phosphate-buffered saline (PBS). At the time of sacrifice, whole blood was collected by puncture of the sinus retro-orbital vein prior analgesia with oxybuprocaine chlorhydrate (Fig. 1).

### IFN-γ ELISpot Assay on PBMCs and spleens

ELISPOT was performed according to BD Biosciences manufacturer instructions (BD ELISPOT Mouse IFN-γ ELISPOT Set cod. 551,083). 5 × 10^5^ splenocytes or 2 × 10^5^ PBMCs from pooled samples were counted, plated in each well and stimulated with 10ug/ml of single peptides used for the immunization and incubated for 24-26 h. As negative and positive control, peptide diluents PBS and 5ug/ml of phorbol myristate acetate (PMA, Sigma-Aldrich) were used respectively. Plates were read with an AID EliSpot Reader Systems (AID GmbH, Strassberg, Germany). The results were calculated as spot forming counts as a mean of a duplicate count from the specific antigen stimulation minus the negative control.

### Statistical analysis

Comparison between individual data points were performed with the unpaired two-sided Student’s *t*-test and ANOVA, as appropriate. Normally distributed data were represented as mean ± S.E.M. Two-way ANOVA and Bonferroni *post-hoc* analysis were used to examine the significance of differences among groups. All p values were two-tailed and considered significant if less than 0.05.

## Results

### Prediction and design of heteroclitic peptides

The HPV-E7 and Trp2 TAAs were subjected to global protein BLAST against the viral as well as the microbiota sequences within the GeneBank non-redundant protein database. The search returned only microbiota sequences sharing homology with the TAAs. In particular, HPV-E7 RAHYNIVTF peptide shows 100% homology with a peptide derived from *Listeria monocytogenes* Consequently, in order to simulate a viral mimicry of the wild type Trp2 and HPV E7 epitopes, heteroclitic peptides (hPep) were designed modifying the residues which directly interact with the H-2Db major histocompatibility complex – I (position 3 and 5) or with the T cell receptor (TCR) (position 4) [27]. In particular, substitutions were selected according to improved or reduced binding affinity to the H-2Db molecule as predicted by NetMHCpan 4.1 software. Regarding the HPV-E7 TuA, the wt RAHYNIVTF peptide has a predicted binding affinity of 38.51 nM and the selected heteroclitic peptides were the p3 RA**I**YNIVTF and p4 RAH**A**NIVTF peptides with an increased binding (15.02 and 29.05 nM, respectively) and the heteroclitic p5 RAHY**H**IVTF peptide with a reduced binding (1628.54 nM). Regarding the Trp2 TuA, the wt SVYDFFVWL peptide has a predicted binding affinity of 9502.51 nM. The selected heteroclitic peptides were the p3 SV**C**DFFVWL with a reduced binding (18632.6 nM), the p4 SVY**A**FFVWL with a similar binding (8459.83 nM) and the p5 SVYD**N**FVWL with an increased binding (252.08 nM). Interestingly, the modification of the p4, which does not directly interact with the H-2Db molecule, had an impact on the binding of the epitope. This was evident for both epitopes, but it was more relevant for the HPV-E7 TuA with a 24.5% increased binding affinity (from 38.51 to 29.05 nM for the p4 RAH**A**NIVTF peptide).(Suppl Fig. 1).

### Structure modelling and molecular docking

**Fig. 2:**
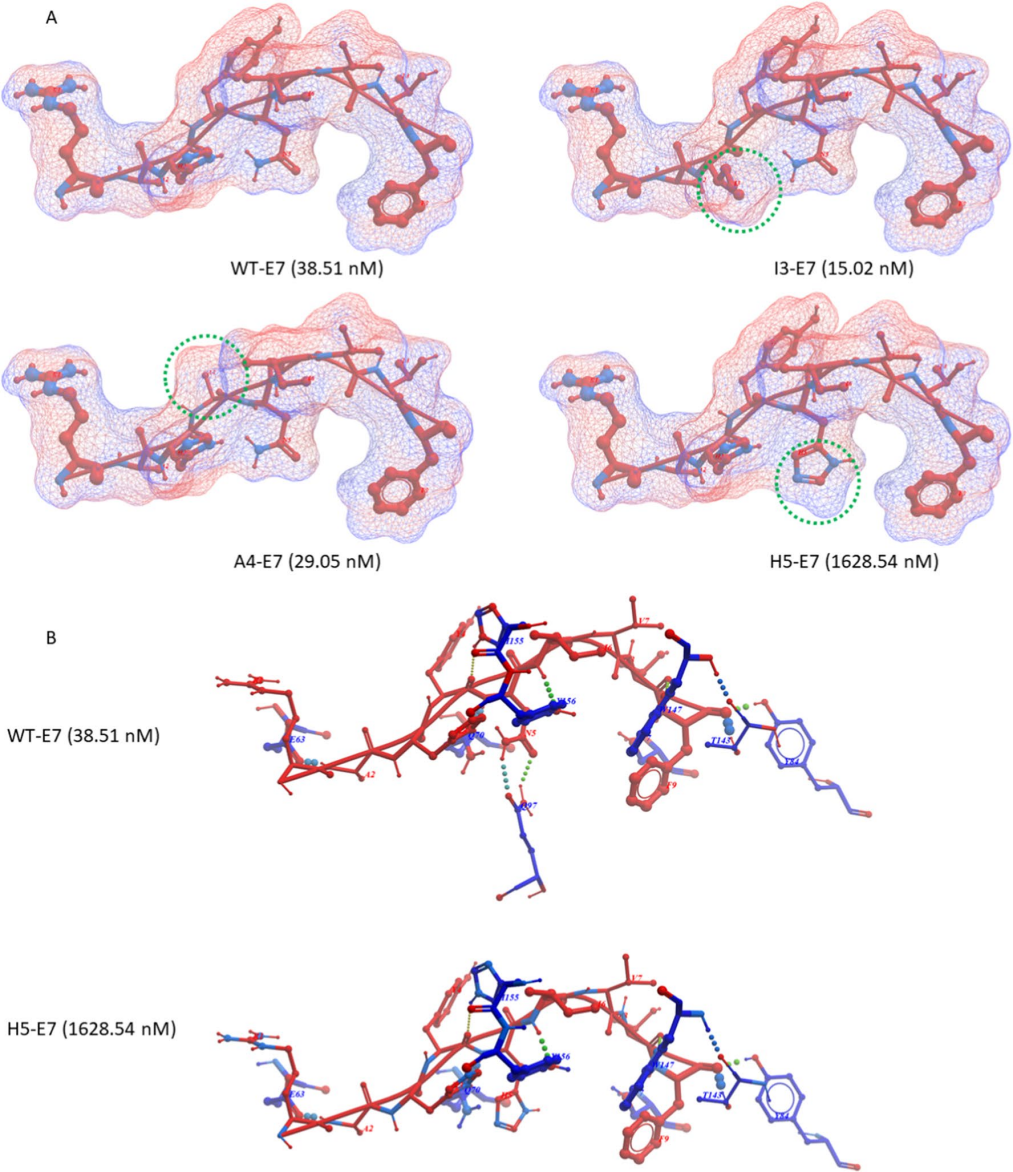
Structural predicted conformation of E7 peptides. **A** The conformation of the wt and hPep peptides bound to the H2-Db molecule is shown. The amino acid substitutions in each hPep is highlighted by the green circle. The predicted affinity value for each peptide is indicated in nanomolarity. **B** The differential hydrogen bonds between the peptide and the H2-Db molecule is indicated for the wt and the H5 hPep peptides

The impact of the aminoacid substitutions on the structure of the heteroclitic peptides as well as the interaction with the H2-Db molecule, was assessed by structure modelling and molecular docking. The modelling confirmed the prediction values for both E7 and Trp2 peptides. Indeed, the substitutions of the H (His) with a I (Ile) at the anchor position 3 of the E7 peptide (heteroclitic I3-E7 peptide) or of the Y (Tyr) with an A (Ala) at the TCR-binding position 4 of the E7 peptide (heteroclitic A4-E7 peptide) do not produce a significant conformational change at contact points with the H2-Db molecule. However, the conformation of the TCR-binding domain is considerably modified in the heteroclitic A4-E7 peptide, suggesting a lack of cross-reactive T cell response. On the contrary, the substitution of the N (Asn) with a H (His) at anchor position 5 of the E7 peptide (heteroclitic H5-E7 peptide) produces a dramatic conformational change with the loss of hydrogen bonds to the Q_97_ residue in the groove of the H2-Db molecule. Consequently, the binding affinity of the heteroclitic H5-E7 peptide is 42.28 fold lower than the wt (Fig. 2A and B).

**Fig. 3:**
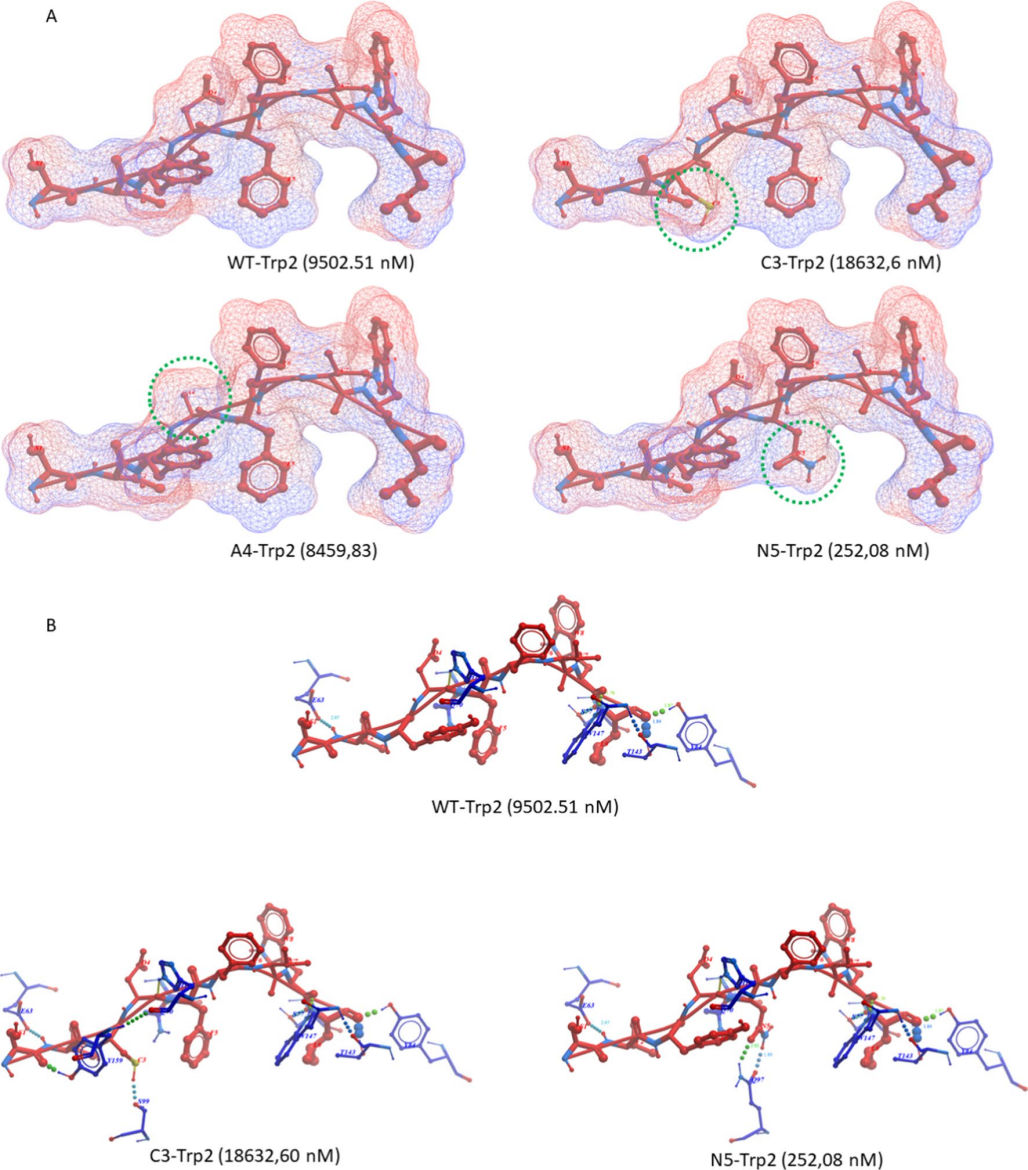
Structural predicted conformation of Trp2 peptides. **A** The conformation of the wt and hPep peptides bound to the H2-Db molecule is shown. The amino acid substitutions in each hPep is highlighted by the green circle. The predicted affinity value for each peptide is indicated in nanomolarity. **B** The differential hydrogen bonds between the peptide and the H2-Db molecule is indicated for the wt and the C3 and N5 hPep peptides

Likewise, the substitution of the Y (Tyr) with a C (Cys) at anchor position 3 of the Trp2 peptide (heteroclitic C3-Trp2 peptide) produces a conformational change with the formation of additional hydrogen bonds to the Y_159_ and S_99_ residues in the groove of the H2-Db molecule. This conformation change is not favorable and the binding affinity of the heteroclitic C3-Trp2 peptide is twofold lower than the wt. The substitution of the D (Asp) with an A (Ala) at the TCR-binding position 4 of the Trp2 peptide (heteroclitic A4-Trp2 peptide) do not produce a significant conformational change at contact points with the H2-Db molecule. However, the conformation of the TCR-binding domain is considerably modified in the heteroclitic A4-Trp2 peptide, suggesting a lack of cross-reactive T cell response. On the contrary, the substitution of the F (Phe) with a N (Asn) at anchor position 5 of the Trp2 peptide (heteroclitic N5-Trp2 peptide) produces a conformational change with the formation of a hydrogen bond to the Q_97_ residue in the groove of the H2-Db molecule. This conformation change is favorable and the binding affinity of the heteroclitic N3-Trp2 peptide is 37.7 fold higher than the wt (Fig. 3A and B).

### In vitro analysis of peptide binding affinity and stability to H-2 Db molecule

In order to experimentally confirm the binding and stability of heteroclitic peptides to H-2 Db molecule, the H-2 Db positive RMA-S cell line was loaded with a sub-optimal concentration of 1 μM of peptides. The results confirmed that the minimal structural conformation change of I3-E7 and A4-E7 peptides allows a binding to the H2-Db molecule equivalent or higher than the wt. On the contrary, the dramatic structural conformational change of H5-E7 peptide induces a complete loss of binding to the H2-Db. Likewise, the C3-Trp2 and A4-Trp2 peptides have comparable structure with the wt and show an equivalent binding to the H2-Db molecule; instead, the N5-Trp2 peptide has a more fitted conformation and binds to the H2-Db much better than the wt (Additional file 1: Figure S2).

### Effect of short-term memory immunity on tumor growth.

Animals were immunized in a preventative setting with either the wt or the heteroclitic E7 and Trp2 peptides. At the end of the full vaccination protocol, animals were challenged with 5 × 10^5^ TC1 or 1 × 10^4^ B16 tumor cells, respectively.

**Fig. 4:**
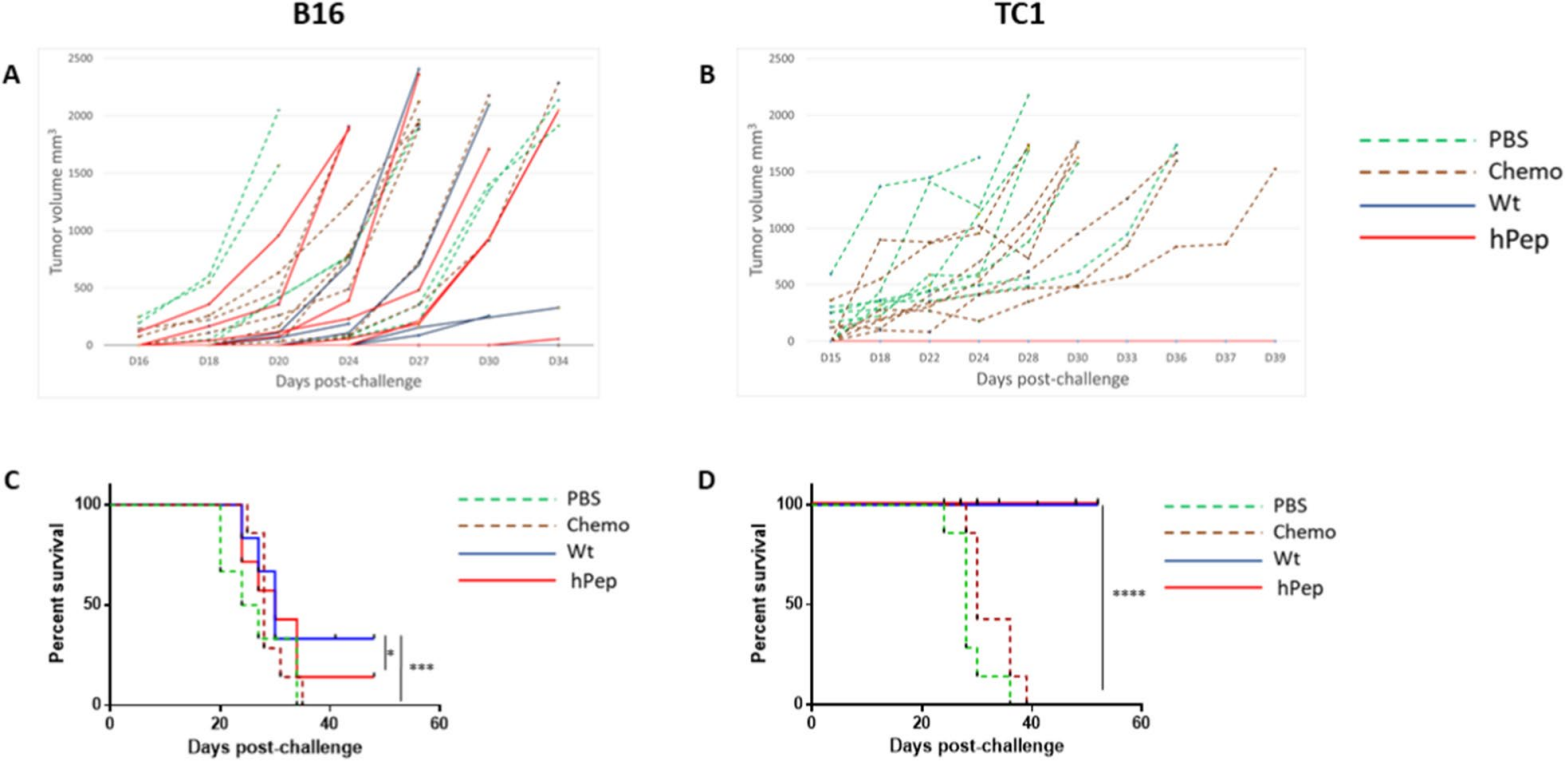
Experimental animal short-term immunization. Mice were treated as described. The curve of tumor growth for the B16 (**A**) and TC1 (**B**) is indicated for each individual animal. Experiments were stopped when all animals in the control groups reached the threshold of 1500 mm^3^. The Kaplan–Meyer curves for the B16 (**C**) and TC1 (**D**) is indicated

The effect of the preventative vaccination was able to significantly delay tumor growth in animals vaccinated with wt or the heteroclitic Trp2 peptides and challenged with B16 tumor cells. Indeed, after 34 days post-challenge, only animals in the vaccinated groups were still alive (2/6, 33.3% in the wt group; 1/7, 14% in the heteroclitic group) (Fig. 4A and C).

Strikingly, in the TC1 challenge experiment, the preventative vaccination with either the wt or the heteroclitic peptides was able to completely inhibit the tumor growth up to day 34, when the last animals in the control groups were euthanized (Fig. 4B, D). Only two animals in the wt groups showed tumor growth very late, at day 42 and day 51 post-challenge, without reaching the cut-off for the sacrifice at the end of the follow up post-challenge (day 60) (data not shown).

### Immunological correlates in the preventive short-term setting

**Fig. 5:**
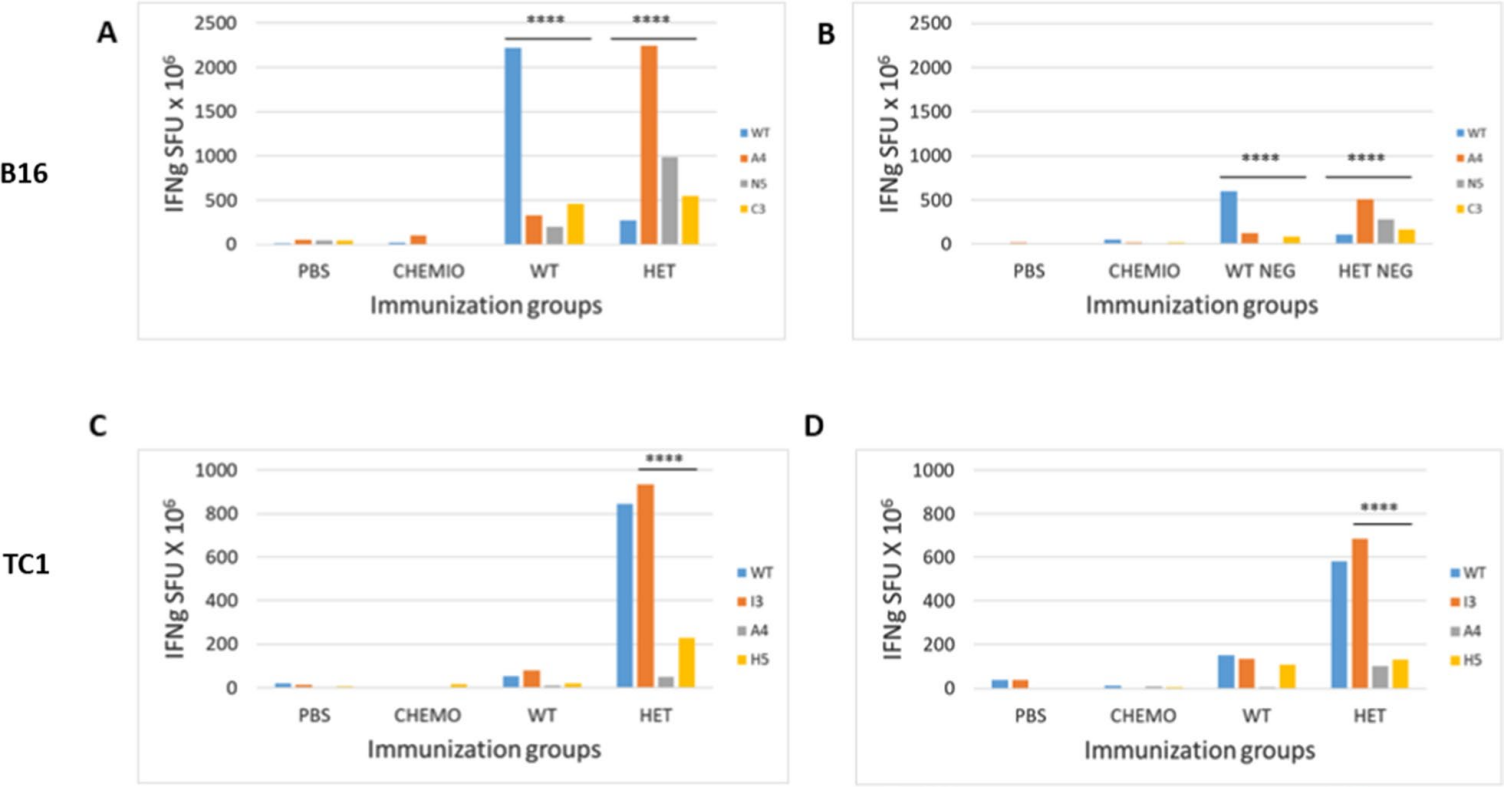
Immunological evaluation in the short-term immunization. Blood samples were drawn during the experiment from retro-orbital veins and PBMCs were isolated. Interferon-γ (IFN-γ) secreting T cells were evaluated in vitro after O/N re-stimulation with individual peptides. Pool of samples were used for each experimental groups. **A** and **B** B16 group; **C** and **D** TC1 group. *SFU* IFN-γ spot forming units

In order to assess the immune correlates of the results in the preventive short-term setting, PBMCs were collected from animals during the experiment. In the B16 experimental group, at the end of the vaccination protocol the immune response induced in the wt and the heteroclitic groups was strong and comparable, although the cross-reactivity was evident but low. Among the heteroclitic peptides, the A4-Trp2 was immunodominant and the low cross-reactivity with the wt confirmed the poor homology in the conformation of the TCR-binding domains. The levels of immune response in both groups faded away during the experiment but remained significantly high, although it was unable to efficiently control the tumor growth. Nevertheless, the better control was observed in the wt group, confirming that low level of immune cross-reactivity induced by the heteroclitic peptides (Fig. 5A and B).

On the contrary, in the TC1 experimental group, the heteroclitic E7 peptides induced an immune response much more potent than the wt with a strong cross-reactivity. Among the heteroclitic peptides, the I3-E7 was immunodominant and the cross-reactivity with the wt confirmed the identical conformation in the TCR-binding domains. Such levels of immune response persisted after the tumor challenge throughout the experiment, supporting the elicitation of a strong anti-tumor T cell activity able to inhibit and control the tumor growth. In the wt group was interesting to observe that the tumor challenge was able to boost the immune response against the vaccine wt and heteroclitic peptides (Fig. 5C and D).

### Effect of long-term memory immunity on tumor growth

**Fig. 6:**
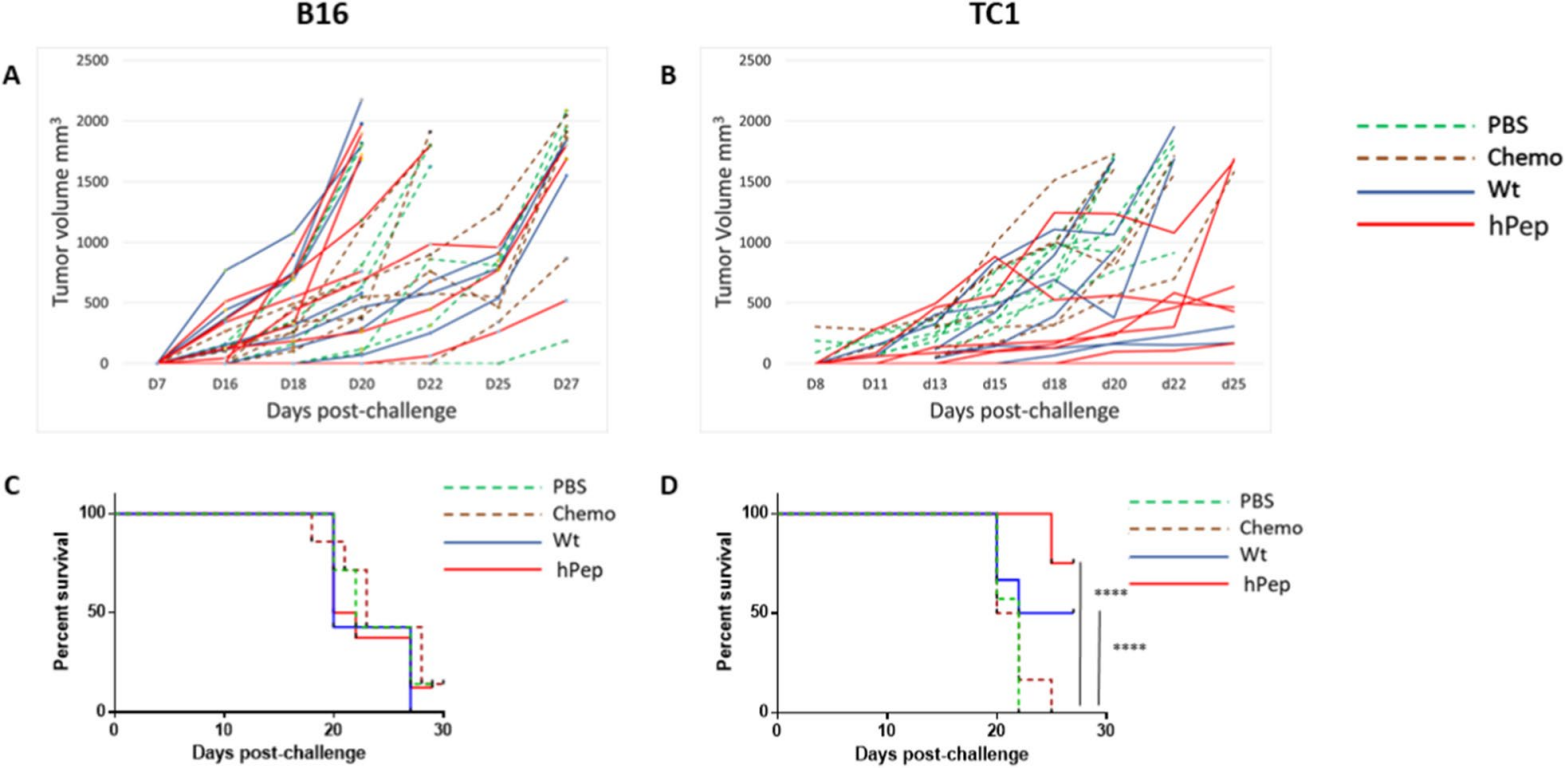
Experimental animal long-term immunization. Mice were treated as described. The curve of tumor growth for the B16 (**A**) and TC1 (**B**) is indicated for each individual animal. Experiments were stopped when all animals in the control groups reached the threshold of 1500 mm^3^. The Kaplan-Meyer curves for the B16 (**C**) and TC1 (**D**) is indicated

In order to assess the anti-tumor efficacy of the long-term memory induced by the vaccination, animals were left free of active immunization for 2 months. Animals were then challenged with 5 × 10^5^ TC1 or 1 × 10^4^ B16 tumor cells, respectively. Results showed that the anti-tumor immune protection was still present but at lower magnitude compared to the short-term immunity. Indeed, in the B16 model, already at day 27 post-challenge all animals in the wt group reached the threshold for euthanasia as for animals in the control groups. On the same day, only one animal was still alive in the heteroclitic group together with one animal in each of the two control groups (Fig. 6A and D). Similarly, in the TC1 model, the long-term immunity was unable to control tumor growth as for the short-term immune response. Nevertheless, compared to controls, tumor growth was significantly delayed in both the wt and heteroclitic groups with a significant statistical improvement in the latter group. Indeed, at 25 days post-challenge when all the animals in the two control groups were euthanized, 50% and 75% animals in the wt and heteroclitic groups were still alive, respectively (Fig. 6B and D).

The head-to-head comparison between the effects in both tumor models of the short- and long-term immunity clearly shows that the latter, although at lower levels, was still able to significantly delay the tumor growth (Additional file 1: Figure S3).

### Immunological correlates in the preventive long-term setting

**Fig. 7:**
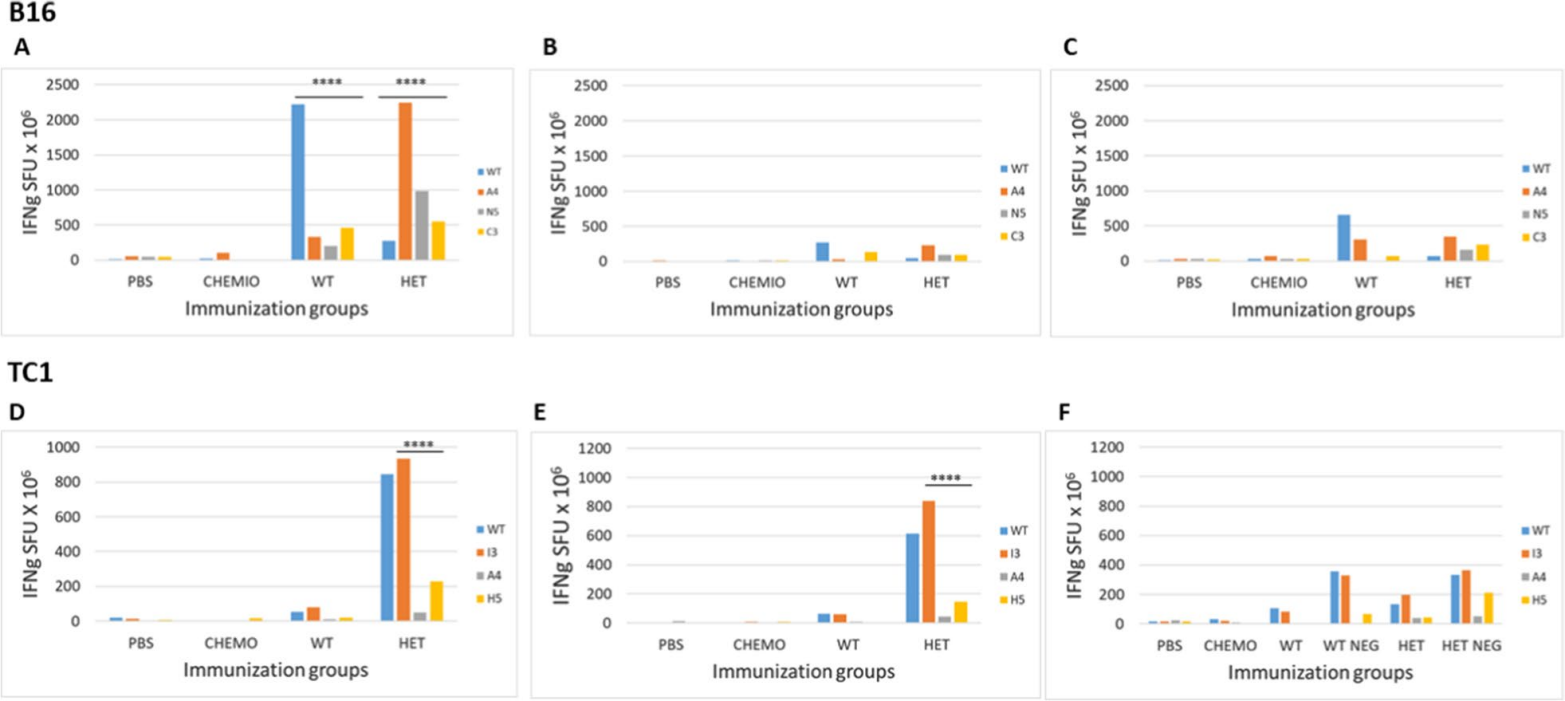
Immunological evaluation in the long-term immunization. Blood samples were drawn during the experiment from retro-orbital veins and PBMCs were isolated. Interferon-γ (IFN-γ) secreting T cells were evaluated in vitro after O/N re-stimulation with individual peptides. Pool of samples were used for each experimental groups. **A**–**C** B16 group; **D**–**F** TC1 group. *WT NEG* animals in the WT group with no sign of tumor growth, *HET NEG* animals in the Heteroclitic group with no sign of tumor growth

In order to assess the immune correlates of the results in the preventive long-term setting, PBMCs were collected from animals during the experiment. In the B16 experimental group, at the end of the vaccination protocol the immune response induced in the wt and the heteroclitic groups was strong and comparable, although the cross-reactivity was evident but low. Among the heteroclitic peptides, the A4-Trp2 was immunodominant and the low cross-reactivity with the wt confirmed the poor homology in the conformation of the TCR-binding domains. The levels of immune response in both groups faded away during the experiment but remained significantly high, although it was unable to efficiently control the tumor growth. Nevertheless, the better control was observed in the wt group, confirming that low level of immune cross-reactivity induced by the heteroclitic peptides (Fig. 7A–C).

In the TC1 experimental group, the immunity was still at the same level 2 months after the end of the vaccination protocol and right before the tumor challenge. Similar to what observed at the end of the vaccination, the heteroclitic E7 peptides induced an immune response much more potent than the wt with a strong cross-reactivity and the I3-E7 peptide was immunodominant. Such levels of immune response persisted but showed a progressive reduction after the tumor challenge throughout the experiment, although this was sufficient to control the tumor growth in a good percentage of animals. Interestingly, a statistical difference in immune response was observed in vaccinated animals with no sign of tumor growth, compared to those with measurable tumors (Fig. 7F). Also in this experimental setting, it was confirmed that the tumor challenge in the wt group was able to boost the immune response against the vaccine wt and heteroclitic peptides (Fig. 7D–F).

## Discussion

In the present study we aimed at verifying whether an established T cell memory specific for antigens sharing sequence and structural similarity with a TuA, as mimicry of MoAs, may control tumor growth in an animal model.

A BLAST search failed to identify viral peptides with high homology to the HPV-E7 and the Trp2 TuAs. Therefore, to simulate viral peptides encountered during the lifetime, they have been modified in the positions 3 and 5, which directly interact with the H-2Db major histocompatibility complex—I, or position 4 which directly interact with the T cell receptor (TCR). The heteroclitic peptides have been selected with different binding affinity to the H2-Db and different structure compared to wt TuAs.

For the HPV-E7 TuA the heteroclitic peptides with H3 → I (I3-E7 peptide) and the Y4 → A (A4-E7 peptide) substitutions were selected with an increased binding; the one with N5 → H (H5-E7 peptide) was selected with a reduced binding. For the Trp2 TuA the heteroclitic peptide with Y3 → C (C3-Trp2 peptide) was selected with a reduced binding; the one with the D4 → A (A4-Trp2 peptide) substitution was selected with a similar binding; the one with F5 → N (N5-Trp2 peptide) was selected with an increased binding. The bionformatic structural modelling confirmed the predicted binding values and showed a comparable similarity in the TCR-binding domain between the heteroclitic and the corresponding wt peptides. The only exceptions were the A4-E7 and the A4-Trp2 peptides, suggesting a lack of cross-reactive T cell response with the corresponding wt peptides.

All the predictions were fully confirmed by binding and stability assay using the TAP-deficient H-2 Db positive RMA-S cell lines.

The preventive vaccination setting was designed immunizing mice either with the wt TuAs or with the mix of the heteroclitic peptides; the challenge with the corresponding tumor cells was performed either at end of the vaccination protocol (short-term) or after 2 months (long-term). In particular, the latter experimental design allows the establishment of a long-term immunological memory, corresponding to an interval of 8–10 years in human life.

The results clearly show that the short-term immunity induced by the wt and the heteroclitic peptides is extremely powerful in containing tumor growth in the TC1 tumor model with 100% survival in both groups. The preventive efficacy in the B16 tumor model is lower with a survival of 33% in the wt group and 14% in the heteroclitic experimental groups. However, considering that this tumor is extremely “immunologically cold” with a very poor lymphocyte infiltration and hard to be controlled by the anti-tumor T cell immunity, the observed survival is to be considered remarkable.

Even more important are the results in the long-term immunization setting. Indeed, although the protective effect was reduced, the established memory T cell was still able to greatly control tumor growth in TC1 tumor model. Indeed, 75% of animals (6/8) were still alive in the heteroclitic group, with 2 tumor-free animals, and 43% (3/7) in the wt group, with 1 tumor-free animal. Survival in the B16 tumor model was much lower with a single animal still alive in the heteroclitic group at the end of the experiment. But this observation might be confounding given that one animal was still alive also in each of the control groups.

The results of tumor growth and survival were fully supported by the assessment of T cell immune responses specific for the vaccine peptides. Indeed, in the TC1 setting, the heteroclitic peptides induced a strong and persisting immune response, prevalently by the I3-E7 peptide, strongly cross-reacting with the wt. On the contrary, in the BC16 setting, the immune response induced by the heteroclitic peptides, prevalently by the A4-Trp2 peptide, was persisting but poorly cross-reacting with the wt. This latter observation could additionally explain the limited anti-tumor efficacy of the induced T cells response.

Our study shows for the first time that an immunological memory elicited by heteroclitic peptides closely resembling a TuA is cross-reactive and able to inhibit or delay tumor growth. In our setting, such heteroclitic peptides can be considered as mimicking viral antigens encountered by the immune system during the lifetime (“*molecular mimicry*”). The effect is extremely powerful if the tumor would start growing right after the “natural viral immunization”. But, most importantly, it is still observed in mice after a remarkable period corresponding to 8–10 years in the human life. As predictable, the potency of such effect is directly correlated to the sequence and structural homology between the epitopes as well as to the responsiveness of the tumor to immunotherapy.

In conclusion, this is the first experimental demonstration that the previous exposure to viral epitopes may result in the establishment of a bi-specific anti-viral/anti-cancer T cell memory if a cancer would develop during the lifetime presenting, by chance, a TAA sharing sequence and conformation similarities with the viral epitope. This may ultimately represent a relevant selective advantage for cancer patients. Moreover, the homolog viral antigens are non-self which do not suffer from the immunological tolerance and would be a totally new set of antigens for developing a novel and more potent preventive/therapeutic anti-cancer vaccine strategy.

## Supplementary Information

**Additional file 1: Fig. S1**. Predicted affinity of wt and hPep peptides. The affinity to H2-Db were predicted by NetMHCpan 4.1 for wt and heteroclitic peptides. The affinity (Aff) values are expressed in nanomolarity (nM). The selected hPep peptides are indicated for both E7 and Trp2 peptides. **Fig. S2**. Experimental affinity of wt and hPep peptides. Binding to H2-Db molecule was assessed in TAP-deficient RMA-S cells loaded with the indicated peptides. Mean fluorescence intensity at flow cytometer indicates binding levels of peptides to HLA molecules. **Fig. S3.** Comparison of short-term and long-term immunization. The curve of tumor growth in the short-term and long-term immunization experiments for the B16 (A) and TC1 (B) is indicated for each individual animal. The Kaplan-Meyer curves for the B16 (C) and TC1 (D) is indicated.

## Data Availability

Data and material will be deposited and publicly available.
