# Supplementary material for: Long-term memory T cells as preventive anticancer immunity elicited by TuA-derived heteroclitic peptides
Source: J Transl Med. 2021 Dec 24;19:526. doi: 10.1186/s12967-021-03194-6 (PMC8709997; doi:10.1186/s12967-021-03194-6)
Supplement: Supplementary file 1 — Additional file 1: Fig. S1. Predicted affinity of wt and hPep peptides. The affinity to H2-Db were predicted by NetMHCpan 4.1 for wt and heteroclitic peptides. The affinity (Aff) values are expressed in nanomolarity (nM). The selected hPep peptides are indicated for both E7 and Trp2 peptides. Fig. S2. Experimental affinity of wt and hPep peptides. Binding to H2-Db molecule was assessed in TAP-deficient RMA-S cells loaded with the indicated peptides. Mean fluorescence intensity at flow cytometer indicates binding levels of peptides to HLA molecules. Fig. S3. Comparison of short-term and long-term immunization. The curve of tumor growth in the short-term and long-term immunization experiments for the B16 (A) and TC1 (B) is indicated for each individual animal. The Kaplan-Meyer curves for the B16 (C) and TC1 (D) is indicated. [file 12967_2021_3194_MOESM1_ESM.pptx]

## Slide 1
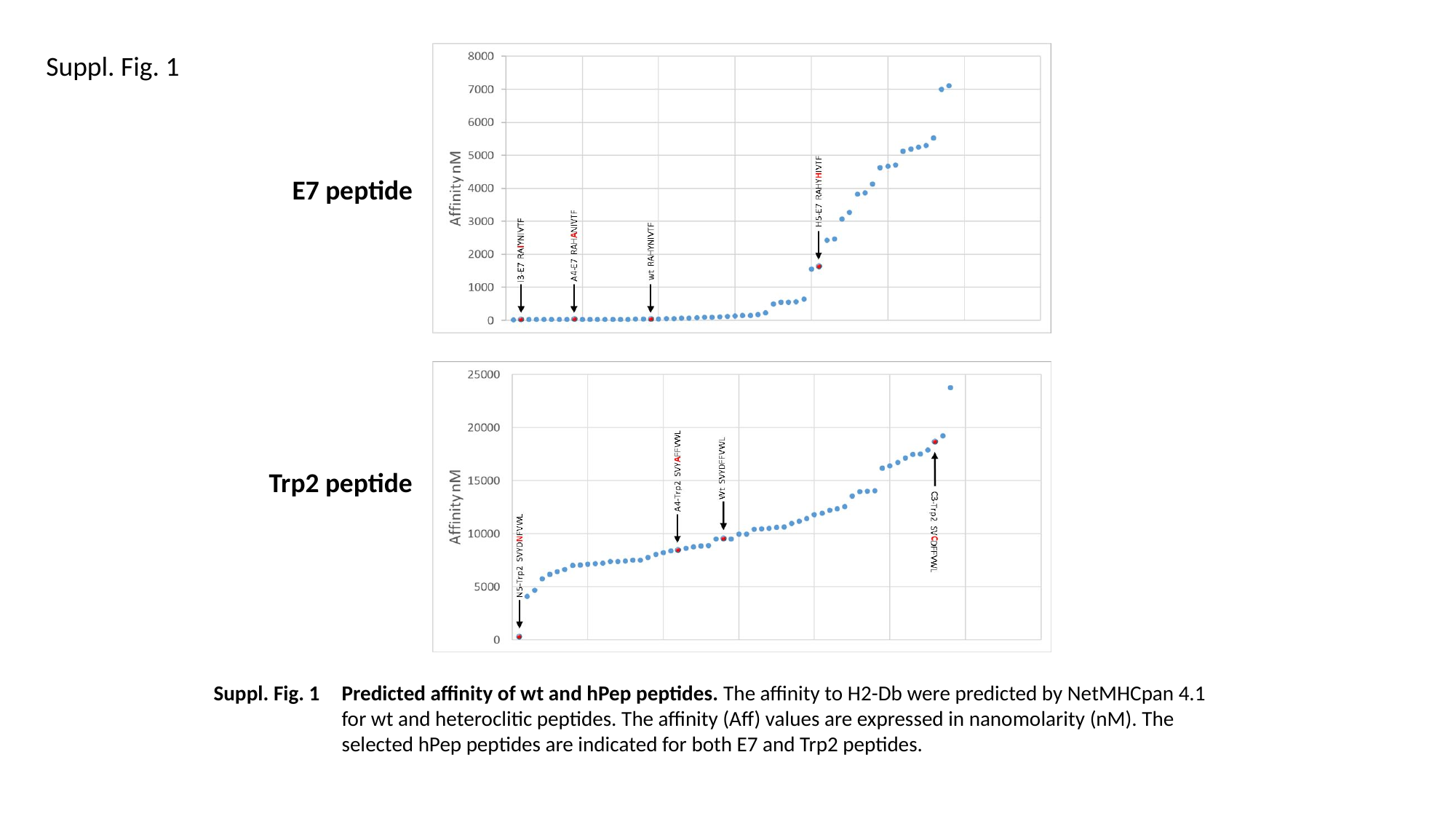

Suppl. Fig. 1
E7 peptide
Trp2 peptide
Suppl. Fig. 1	Predicted affinity of wt and hPep peptides. The affinity to H2-Db were predicted by NetMHCpan 4.1 for wt and heteroclitic peptides. The affinity (Aff) values are expressed in nanomolarity (nM). The selected hPep peptides are indicated for both E7 and Trp2 peptides.

## Slide 2
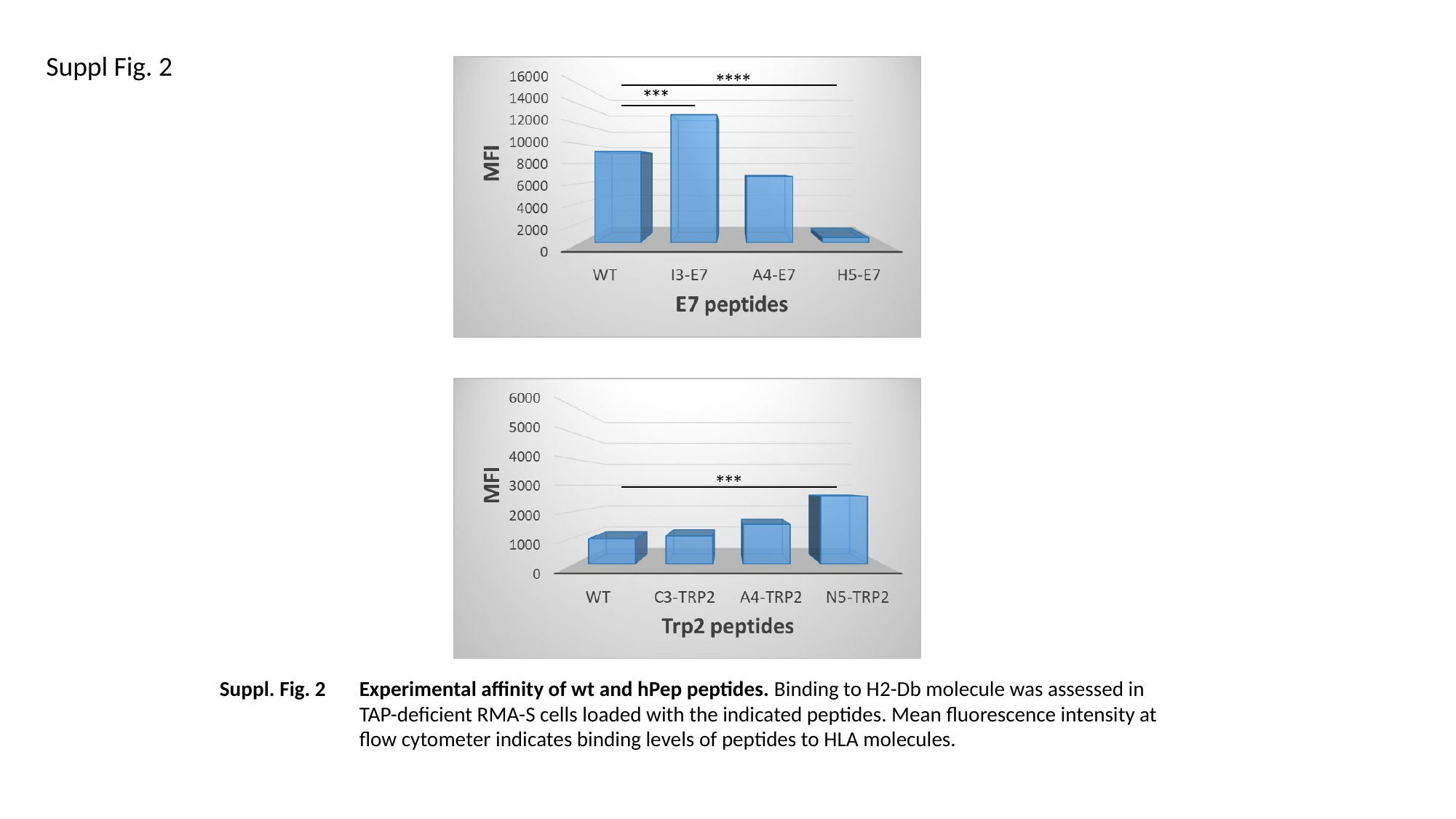

Suppl Fig. 2
Suppl. Fig. 2	Experimental affinity of wt and hPep peptides. Binding to H2-Db molecule was assessed in TAP-deficient RMA-S cells loaded with the indicated peptides. Mean fluorescence intensity at flow cytometer indicates binding levels of peptides to HLA molecules.

## Slide 3
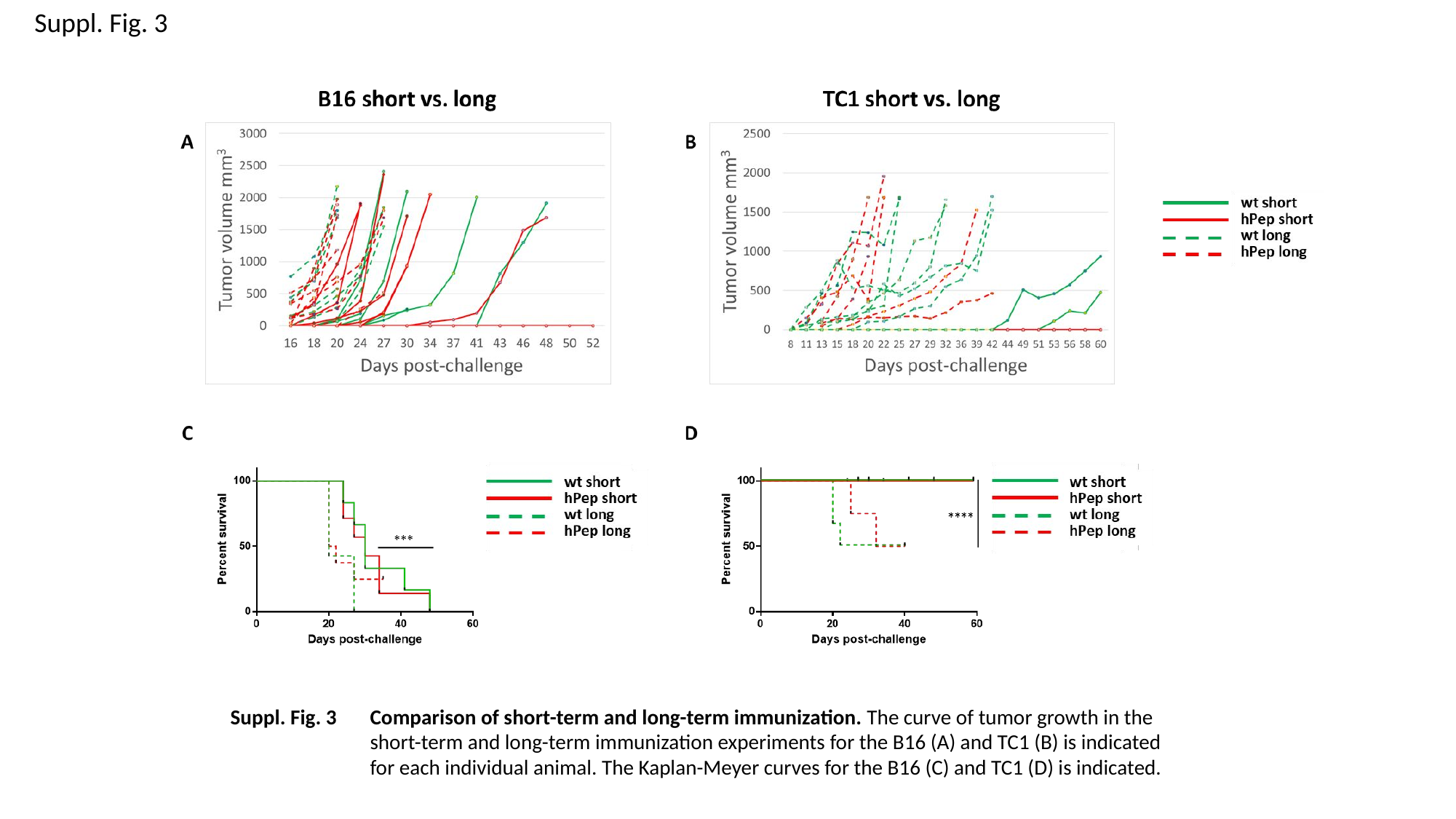

Suppl. Fig. 3
Suppl. Fig. 3	Comparison of short-term and long-term immunization. The curve of tumor growth in the short-term and long-term immunization experiments for the B16 (A) and TC1 (B) is indicated for each individual animal. The Kaplan-Meyer curves for the B16 (C) and TC1 (D) is indicated.
